# Optimization of degradation conditions and elucidation of novel biodegradation pathways for sulfamonomethoxine by a novel *Bacillus* strain

**DOI:** 10.1128/aem.01329-25

**Published:** 2025-08-12

**Authors:** Xiujuan Wang, Jingtong Li, Chunyan Chen, Zifeng Luo, Yuwan Pang, Hongxing Tu, Xiaojun Lin, Cuifen Long, Qianyi Cai, Zebin Wei, Jinrong Qiu

**Affiliations:** 1 South China Institute of Environmental Sciences, MEE, Guangzhou, Guangdong, China; 2 State Environmental Protection Key Laboratory of Water Environmental Simulation and Pollution Control, Guangzhou, Guangdong, China; 3 College of Agriculture, Yangtze University, Jingzhou, Hubei, China; 4 Jinan University 47885, Guangzhou, China; 5 Institute of Agricultural Resources and Environment, Guangdong Academy of Agricultural Sciences 117866, Guangzhou, China; 6 College of Natural Resources and Environment, South China Agricultural University 12526, Guangzhou, China; 7 Guangzhou Municipal River and Channel Monitoring Center, Guangdong, Guangzhou, China; Universidad de los Andes, Bogotá, Colombia

**Keywords:** sulfamonomethoxine (SMM), *Bacillus* sp. DLY-11, biodegradation, condition optimization, biodegradation pathway

## Abstract

As a commonly used sulfonamide antibiotic, the efficient reduction of sulfamonomethoxine (SMM) residue in the environment is a critical issue that urgently needs to be addressed. However, there is limited understanding of the microbial conditions needed for efficient SMM degradation and its mechanisms. Therefore, this study screened a new strain, *Bacillus* sp. DLY-11, from swine manure compost with significant SMM degradation capability, and utilized response surface methodology (RSM) based on Box-Behnken design to optimize the degradation conditions. The results showed that under conditions of a 5% inoculation volume, a temperature of 59.1°C, a pH value of 7.10, and 0.45 g/L MgSO_4_, strain DLY-11 could degrade 98.8% of 20 mg/L SMM within 48 h. Product analysis identified six potential transformation products and proposed two potential biodegradation pathways of SMM, including C-N bond cleavage, hydroxylation, and SO_2_ release. Particularly, we discovered a novel degradation pathway that has not been reported before. This study not only introduced a new strain for efficient SMM degradation but also optimized conditions and revealed new degradation pathways. These findings addressed gaps in bacterial SMM degradation pathways, offering theoretical and technical support for bioremediating antibiotic pollutants in animal husbandry.

**IMPORTANCE:**

The discovery of a new *Bacillus* sp., strain DLY-11, from aerobically composted swine manure offers significant environmental benefits by efficiently degrading 98.8% of 20 mg/L sulfamonomethoxine (SMM) within 48 hours under optimal conditions (5% inoculation volume, 59.1°C, pH 7.10, 0.45 g/L MgSO4). This strain introduces a new tool for reducing SMM antibiotic pollution and reveals a novel degradation pathway, enhancing our understanding of SMM biodegradation mechanisms and supporting targeted bioremediation strategies.

## INTRODUCTION

Sulfonamide antibiotics are a broad-spectrum class of antibiotics widely used in human disease treatment and to promote growth in livestock. However, due to the incomplete metabolism (less than 10%) of antibiotics in humans and animals, approximately 30%−90% of the antibiotics are excreted into the environment through feces and urine (1, 2), posing a long-term threat to human health and ecosystems (3, 4). Although sulfonamide antibiotics can degrade in the environment due to factors such as light, temperature, microorganisms, and other influences, the degradation rate is very slow, leading to prolonged drug residue times (5, 6). Current research has detected sulfonamide antibiotics in livestock farms (7), natural rivers (8), domestic wastewater (9), groundwater (10), soil (11), and even residential tap water (12). Long-term environmental exposure to residual sulfonamide antibiotics will lead to the emergence and enrichment of antibiotic-resistant bacteria and antibiotic resistance genes, not only harming the microbial community structure in ecosystems (13, 14) but also causing risks such as aplastic anemia and genetic toxicity carcinogenicity, severely threatening human health (15). Therefore, it is essential to inactivate or degrade antibiotics before the excretion of animal feces and urine into the environment. Developing an effective method to degrade sulfonamide antibiotics in the environment is urgently needed.

Currently, the techniques for degrading sulfonamide antibiotics mainly fall into non-biological and biological methods. Non-biological methods include adsorption (16), photodegradation (17), and electrochemical oxidation (18). However, these methods are limited by factors such as adsorbents, catalysts, wavelength of ultraviolet light, and the choice of reactor, and the materials and equipment required increase the degradation cost and may introduce new pollutants. By contrast, biological methods are gaining attention due to their adaptability, short growth cycles, low cost, and environmental friendliness (19–22). Biological treatment techniques primarily include natural treatment, anaerobic treatment, aerobic treatment, and their combinations (23–25). Among biological treatment techniques, the effectiveness of antibiotic degradation from high to low is: composting >anaerobic digestion >manure storage >soil (26). Whether it is anaerobic digestion, membrane bioreactor, or aerobic composting for antibiotic degradation, these methods mainly rely on microbial degradation. Research has found that microorganisms degrade sulfonamide antibiotics through multiple pathways, and the specific mechanisms of these pathways are closely related to the bacterial species. For example, Du et al. (27) isolated an antibiotic-degrading strain, *Alcaligenes aquatillis* FA, from the activated sludge of a pharmaceutical plant in China: this strain has a degradation efficiency of 40%–50% for sulfamethoxazinediazine (SMD) and was found to possess *sul2* and *dfrA* genes that confer resistance to sulfonamides (SAs). Xu et al. (28) found that sulfamethoxazole had a degradation efficiency of 83% in the sediment of a natural river at 25°C, with *Bacillus firmus* and *Bacillus cereus* being the main strains involved in this biodegradation. Although degradative strains have been isolated from activated sludge and river sediment, the effectiveness of these strains varies, and key factors influencing degradation efficiency have not been fully optimized and validated. In addition, the byproducts produced during the degradation of sulfonamide antibiotics and their specific mechanisms have not been entirely revealed, particularly in practical production environments such as aerobic composting of swine manure. Therefore, optimizing the parameters for the degradation of sulfonamide antibiotics by degradative strains and conducting in-depth research on the degradation process are of significant theoretical and practical value for their widespread application and better understanding of the degradation mechanisms.

Sulfamonomethoxine (SMM) is a commonly used sulfonamide antibiotic. It is mainly used to prevent and treat bacterial infections in poultry and pigs, serving as a preventive and therapeutic drug in animal husbandry (29). SMM acts by inhibiting the synthesis of folic acid in bacterial cells, thereby suppressing bacterial growth and reproduction (30). However, due to its persistence in the environment and potential ecological risks, research on its environmental impact has increased in recent years (31, 32). Therefore, this study focuses on SMM as the target compound, aiming to isolate and identify a new strain capable of degrading SMM from aerobic composting of swine manure. Through this study, we successfully discovered a new strain, *Bacillus* sp. DLY-11, which has SMM degradation capability. We systematically analyzed the degradation kinetics and characteristics of this strain under various environmental conditions and used response surface methodology (RSM) based on a Box-Behnken design to optimize the degradation conditions. Specifically, we investigated the effects of factors such as temperature, pH, and metal ion concentration on the degradation efficiency of SMM. In addition, by detecting the intermediate and final products during the degradation process, we initially explored the degradation mechanisms of *Bacillus* sp. DLY-11 in degrading SMM. The study not only provided a new degradative strain for effectively degrading SMM but also optimized the relevant conditions and revealed its degradation pathways, offering new insights into the bacterial degradation mechanisms of sulfonamide antibiotics. These findings are expected to provide strong theoretical and technical support for the bioremediation of antibiotic pollutants in animal husbandry.

## MATERIALS AND METHODS

### Experimental materials and chemical reagents

Swine manure samples were collected from a large pig farm in the suburbs of Guangzhou City, and the wood chips used were processing waste from a furniture factory in Dongguan City. The basic properties of the samples are as follows: Swine manure has Total C of 15.3%, Total N of 0.93%, C/N ratio of 16.5, and Moisture content of 53.6%; Wood chips have Total C of 50.8%, Total N of 0.84%, C/N ratio of 60.4, and Moisture content of 4.60%. The required SMM standard (purity >99%) was provided by Shanghai McKinney Biochemical Technology Co., Ltd. (hereinafter referred to as “McKinney Reagent”). Other reagents used in the base medium, such as CaCl_2_, KH_2_PO_4_, MnSO_4_, MgSO_4_, and FeSO_4_, also came from McKinney Reagent. Methanol (chromatography grade), formic acid (98%), and LiChrolut EN solid-phase extraction columns were supplied by Merck, Germany, in Darmstadt. Milli-Q water (resistivity 18.2 MΩ·cm) was prepared using the Millipore water purification system in Billerica, California, USA.

### Effects of aerobic composting on SMM degradation

This experiment used a self-designed aerobic composting device (see Figure B.1), with detailed equipment parameters provided in the supplemental material. The experiment included one treatment method and three replicates. Based on the initial content of SMM antibiotics in swine manure, SMM was added to achieve a preset concentration of 20 mg/kg. The wood chips were immersed in distilled water for 180 minutes before being mixed with swine manure, and the moisture content was adjusted to 65%. The total weight of the compost was 50 kg with a C/N ratio of 25. Aerobic composting was performed using static forced ventilation, with air supplied intermittently from the bottom at a ventilation rate of 0.050 m^3^/min, for 30 minutes every 5 h. Samples were taken every 7 d during the composting process to collect samples from the early, middle, and mature stages of composting. The SMM content was measured to evaluate changes in antibiotic content before and after composting and to calculate the removal efficiency.

### Isolation and identification of SMM-biodegrading bacteria

From the results of aerobic composting of swine manure, the optimal degradation efficiency of SMM was 19.4%, and strains capable of degrading SMM were selected. Through enrichment culture, initial selection and purification, acclimatization, and final selection, the superior new strain DLY-11, highly effective in degrading SMM, was identified. The specific process is detailed in the supplemental material. The strain was inoculated into selective culture media, stained with Gram stain after culturing for 2 d at 30°C, and observed under a scanning electron microscope (33). Physiological and biochemical experiments were conducted based on “Berger’s Manual of Systematic Bacteriology” and “Common Bacterial Systematic Identification Manual” (34, 35). Genetic information was determined by combining 16S rRNA sequencing and genetic analysis, as detailed in the supplemental material.

### Biodegradation kinetics of SMM

The selected strain DLY-11 was inoculated into LB medium and cultured for 12 h under constant temperature and shaking conditions at 30°C and 160 r/min to prepare seed culture. Growth curve analysis was performed to understand the growth characteristics of the strain over time and optimize culture conditions. The strain DLY-11 was inoculated at a 3% inoculation volume into base medium containing 20 mg/L SMM and pH 7. Under the same conditions, the cultures were shaken for 48 h, with samples taken at 0 h, 1 h, 3 h, 6 h, 10 h, 16 h, 24 h, and 48 h to measure the SMM concentration and cell density (OD_600_). Nonlinear regression analysis using a first-order kinetic model was conducted, as detailed in the study by Terzic et al. (36).

### Factors influencing SMM degradation

Based on the degradation kinetics experiment of SMM by strain DLY-11, the study chose a cultivation time of 48 h. Five single-factor influence experiments were conducted, with 200 mL of base medium autoclaved at 121°C for 30 minutes added to each 500 mL glass bottle. Different factors were changed individually: temperature (10°C–40°C), pH (6–9), inoculation volume (1%−5%), type of metal salts (MnSO_4_, MgSO_4_, FeSO_4_, CaCl_2_), and their concentrations (0.1–0.5 g/L) to observe the degradation of SMM. The initial concentration of SMM was set to 20 mg/L, with the appropriate volume of strain DLY-11 culture medium added based on the inoculation volume. Under sterile conditions, the bottles were sealed with rubber stoppers and breathable sterile membrane caps to prevent contamination. The cultures were incubated for 48 h at 30°C and 160 rpm, and the remaining SMM concentration was measured at the end. Before measurement, the supernatant was filtered through a 0.22 µm filter membrane. All experiments were repeated three times.

### Optimization of SMM degradation conditions

The study applied response surface methodology (RSM) and Box-Behnken design (BBD) to optimize the conditions for strain DLY-11 to degrade SMM. The experimental parameters included temperature (X1), pH (X2), and metal ion concentration (X3). The initial concentration of SMM was set to 20 mg/L, with an inoculation volume of 5% in a 500 mL base salt medium, shaken for 48 h at 120 rpm. The specific scheme and parameters of the Box-Behnken design are detailed in the supplemental material, involving three factors, each set at three levels (−1, 0, and +1). BBD can precisely describe linear and nonlinear effects as well as interaction effects because it uses a second-order polynomial model (37). The design evaluates the response at each point within the experimental space and predicts the location of optimal conditions. The experimental setup is presented in Table A.3. Design-Expert 13 software was used for response surface and data analysis. The model’s validity was assessed using ANOVA variance analysis, and the goodness of fit was evaluated using *R2* and adjusted *R2* (38). A quadratic polynomial regression equation was established to represent the mathematical relationship between independent and dependent variables, with the significance of each factor and their interactions being assessed using *P*-values.

### Mechanism of SMM degradation

Under optimized culture conditions, the degradation strain was inoculated into liquid culture medium containing SMM and cultured for 3 d at 30°C. After the culture, the samples were centrifuged, and the supernatant was filtered through a 0.22 µm filter membrane before detecting the SMM degradation products using high-performance liquid chromatography (HPLC, LC-20AD, Shimadzu)-high-resolution hybrid quadrupole time-of-flight mass spectrometry (AB SCIEX X500R Q-TOF). Based on the detection results, the degradation pathway of SMM was inferred, and the functional mechanism of the efficient degradation strain was explained as follows: Removal rate (%) = 100% × (*C*_0_ − *C_t_*)/*C*_0_, where *C*_0_ (mg/L) is the initial concentration of SMM, and *C_t_* (mg/L) is the concentration of SMM in the medium at time *t*.

### Sample preprocessing and instrumental analysis methods

The determination of SMM content was conducted using high-performance liquid chromatography (HPLC, Alliance e2695, Americas), with the internal standard being Sulfamonomethoxine-d4. Before analysis, water samples were filtered through a 0.22 µm pore diameter, 11.7 mm diameter Millipore filter membrane to remove suspended particles. All samples were stored in a dark place at 4°C and underwent solid-phase extraction (16) treatment. The HLB solid-phase extraction columns (6 mL, 200 mg, Waters, USA) were washed with 5 mL methanol and distilled water before sampling, passed through at a flow rate of 3 mL/min, then rinsed with 10 mL distilled water, and dried under a mild airflow. The columns were then eluted with 5 mL methanol, which was evaporated under a nitrogen gas flow at 40°C. The eluate was mixed with 1 mL of methanol and bottled for analysis. The concentration of SMM was determined using HPLC (HPLC, Alliance e2695, Americas), and its degradation metabolites were analyzed using liquid chromatography-quadrupole time-of-flight mass spectrometry (LC-20AD, Shimadzu—AB SCIEX X500R Q-TOF). Detailed steps and parameters of the analysis are provided in the supplemental material.

## RESULTS AND DISCUSSION

### Effects of aerobic composting on SMM degradation

**Fig 1:**
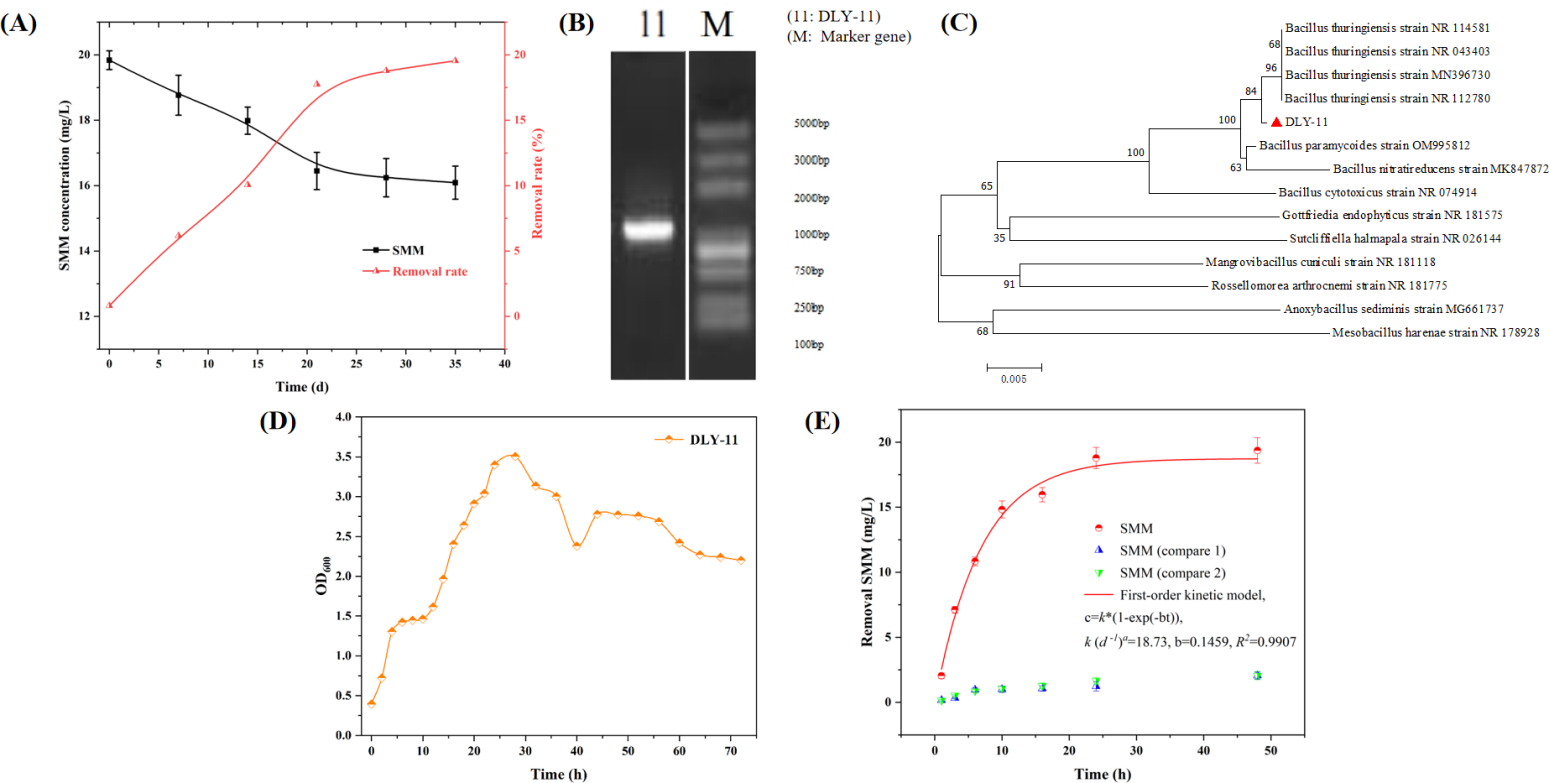
(**A**) Residual concentration of SMM after composting. (**B**) PCR verification results of 16S rRNA of strain DLY-11. (**C**) Phylogenetic tree of strain DLY-11. The phylogenetic tree illustrates the relationships among strains, including DLY-11 and its closely related species, based on the 16S rDNA gene. (**D**) Growth curve of strain DLY-11. (**E**) The change in SMM degradation concentration over time was simulated with a first-order kinetic model, depicting smooth curves. Compare 1: Unvaccinated, Compare 2: Sterilized bacterial cells.

As shown in Fig. 1A, in the 35-day composting experiment, the residual concentration of SMM antibiotics gradually decreased over time, with the degradation effect also strengthening. During the experiment, by monitoring the antibiotic residual concentration at various time points, it was found that the degradation rate of antibiotics exhibited a certain pattern. In the first 20 d, the antibiotic concentration decreased significantly, indicating a fast degradation rate. However, from days 20 to 35, the degradation rate of SMM slowed down, and after 30 d, the degradation efficiency remained largely unchanged. At 35 d, the SMM degradation rate reached 19.5%. Overall, the composting treatment had a moderate effect on the degradation of SMM. This could be due to the fact that during composting, microorganisms not only decompose SMM but also process many other organic substances. These substances may compete with SMM for the active sites of microorganisms or enzymes (39), thereby slowing down the degradation rate of SMM. Although the composting treatment had a generally ordinary effect on SMM degradation, it could be further improved by screening and acclimatizing highly efficient degradative strains. Focusing on microorganisms that are active during the degradation process may lead to the discovery of strains with higher degradation efficiency, thus accelerating the degradation of SMM and optimizing the overall treatment method.

### Strain isolation and identification

Eight strains of bacteria were successfully isolated from the final product of aerobic composting of swine manure, with most showing the ability to reduce the concentration of SMM in LB liquid medium. The DLY-11 strain demonstrated excellent SMM degradation performance and was selected for further research. After multiple acclimatizations, the SMM degradation efficiency of DLY-11 stabilized at approximately 90%. Gram staining revealed that DLY-11 is a Gram-positive rod that typically forms chains, produces spores, and sporocrystals. Based on the analysis of its 16S rRNA gene sequence (GenBank accession no. OQ996970.1) and the phylogenetic tree (see Fig. 1B and 1C, the 16S rRNA sequence length of DLY-11 was 1456 bp, with over 99% homology to *Bacillus* genus bacteria, and it clustered with *Bacillus thuringiensis*. Therefore, DLY-11 was identified as *Bacillus thuringiensis*. This type of bacteria shows great potential in degrading novel pollutants (40). For example, under conditions of 32°C and pH 7.0, *Bacillus thuringiensis* SG4 (KT186610) could degrade 80% of 50 mg/L Cypermethrin within 15 d (41). Another study found that *Bacillus thuringiensis* ZS19 (KF290567) could simultaneously degrade multiple insecticides, including Cyhalothrin (degradation rate 100%), Fenpropathrin (degradation rate 98.3%), and Deltamethrin (degradation rate 92.4%), under conditions of 30°C and a shaking speed of 180 rpm (42). In addition, *Bacillus thuringiensis* BRC-HZM2 (GQ140344) could efficiently degrade Chlorpyrifos, with 50% of the initial concentration degraded within 12 h at 30°C and 230 rpm, and a degradation rate reaching 88.9% after 48 h (43). These studies not only confirmed the excellent performance of DLY-11 in SMM degradation but also provided a solid foundation for its potential in handling novel pollutants.

### *Bacillus sp*. DLY-11 degradation kinetics of SMM

As shown in Fig. 1D, the growth curve of strain DLY-11 indicates the following: from 0 to 2 h, it is the lag phase, where bacteria adapt to the new environment, increase in cell volume, enhance metabolic activity, start dividing, and accumulate enzymes, energy, and intermediate metabolic products. From 2 to 28 h, it is the logarithmic phase, where the number of bacteria increases rapidly at a geometric rate, and the OD_600_ value reaches 3.5 at 28 h. From 28 to 32 h, it enters the stationary phase, where the nutrients in the medium gradually deplete and toxic metabolic products accumulate, leading to a decrease in the rate of bacterial proliferation. After 36 h, it enters the death phase, where the rate of bacterial reproduction is lower than the rate of cellular autolysis and death. When studying the degradation kinetic characteristics of DLY-11 toward SMM, it was found that the method followed a first-order kinetic model (see Fig. 1E), with a correlation coefficient of *R2* = 0.9907 and a degradation rate constant of *k* = 18.73 mg/(L·d) (see Fig. 1E). This finding is consistent with existing research, indicating that biodegradation typically follows a first-order decay model (44–46). For example, Yu et al. (47) isolated a new pregnenolone (PGT) degrading bacterium, *Rhodococcus* sp. HYW, from a pharmaceutical plant, where 99% of PGT was degraded within 1 hour, following a first-order kinetic model with a correlation coefficient *R2* of 0.9992. Similarly, the *P. rettgeri* MB-IIT strain isolated from municipal wastewater treatment plant activated sludge could degrade Triclosan (TCS) under appropriate conditions, showing biodegradation kinetics that also fit a first-order kinetic model, with a correlation coefficient *R2* of 0.9890 (48). These studies demonstrate that the first-order kinetic model is an effective tool for describing the degradation of organic pollutants by bacteria, accurately reflecting the dynamic characteristics of the biodegradation process.

### Single-factor influence on biodegradation of SMM

**Fig 2:**
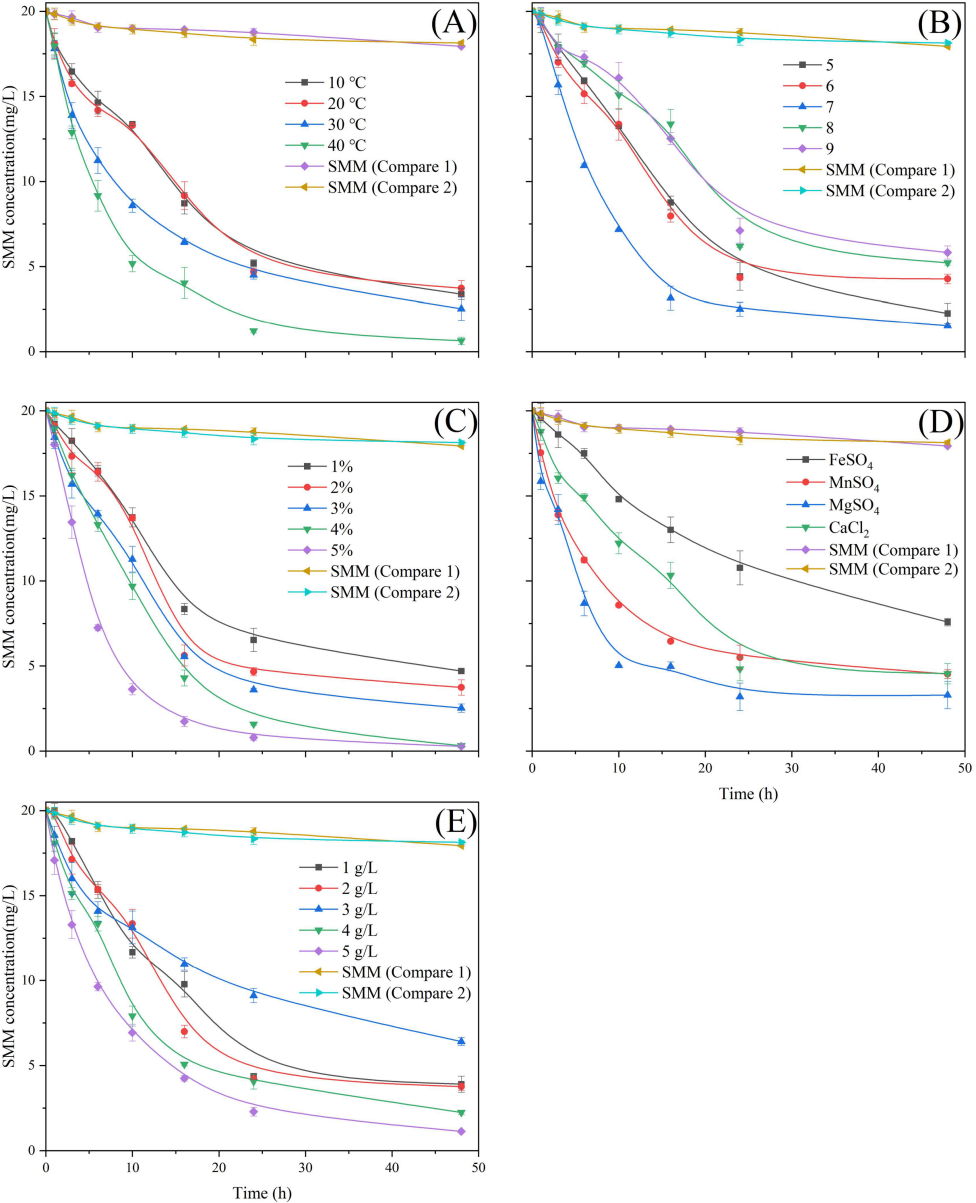
The effect of different treatment conditions on the degradation of SMM by DLY-11. (**A**) Temperature. (**B**) pH value. (**C**) Inoculum size. (**D**) Metallic salt. (**E**) Metal ion concentration. Control 1: Unvaccinated, Control 2: Sterilized bacterial cells.

To improve the degradation efficiency of the DLY-11 strain toward SMM, we investigated several key factors, including temperature, pH, inoculation volume, type of metal salts, and metal ion concentration. Temperature is one of the most important factors influencing the growth and metabolic activity of microorganisms. According to Fig. 2A, the most thorough degradation of SMM occurred when the temperature was between 30°C and 40°C; at 10°C, efficient degradation could not be achieved. At 10°C, the *Bacillus* sp. DLY-11 strain achieved an SMM degradation efficiency of 83.1% within 48 h, while increasing the temperature to 40°C resulted in an SMM degradation rate of 93.9% after 24 h. These results support the notion that elevated temperature composting can accelerate antibiotic degradation (49). Therefore, increasing the cultivation temperature to 40°C can reduce the degradation time, achieving faster degradation and thus optimizing the entire degradation process.

Typically, maintaining an appropriate pH condition is crucial for optimizing the microorganisms’ degradation process of antibiotics (50). The data in Fig. 2B show that the degradation efficiency of the DLY-11 strain varied under different pH conditions. Research findings indicated that between pH 5 and 9, the SMM degradation efficiency followed a trend of first increasing and then decreasing, with the highest efficiency at pH 7. At neutral pH, the SMM degradation rate increased steadily over time, reaching 92.4% after 48 h. By contrast, the degradation effectiveness in alkaline conditions was only around 70%. It can be concluded that a neutral pH is suitable for *Bacillus* sp. DLY-11 biodegradation of SMM, while SMM degradation efficiency decreases in acidic and alkaline conditions. This may be because these pH values are not conducive to microbial growth and metabolism, leading to a decline in their biological activity. This finding is consistent with the conclusion of Wang et al. (51), which indicated that neutral or weakly alkaline pH values (pH 7–8) promote the antibiotic transformation of strain L2-2 and are the most suitable conditions for the degradation of most antibiotics.

Inoculation volume is also an important factor affecting the degradative potential of the target antibiotic and microbial growth. A higher inoculation volume typically provides more degradative microorganisms. With adequate nutrients, this increase in microbial quantity helps produce more degradative enzymes to decompose antibiotics (52). According to the data in Fig. 2C, experiments with an inoculation volume between 4% and 5% showed better degradation performance, with significantly lower residual SMM concentrations and faster degradation rates compared to experiments with inoculation volumes between 1% and 3%. In general, the degradation rate increases with inoculation volume. The study found that at an inoculation volume of 5%, the bacterial degradation efficiency reached 91.3% within 16 h and 98.6% after 48 h, nearly completely degrading SMM. This indicates that under nutrient-rich conditions, a higher inoculation volume can accelerate the DLY-11 strain’s adaptation to the antibiotic environment, thus initiating the degradation process faster. Similarly, a study by Al-Dhabi et al. (53) found that the degradation efficiency of tetracycline increased with inoculation volume. A study by Selvi et al. (54) also found that cephradine degradation efficiency significantly improved based on the bacterial strain inoculation dose and large biomass, with increased antibiotic removal rates and decreased tetracycline concentrations. However, in nutrient-poor conditions, an excessively high inoculation volume can lead to competition among strains, reducing the degradation efficiency of pollutants (48).

Previous studies have shown that metal ions can promote the degradation of antibiotics (55). Some researchers believe that this is because metal ions form coordination reactions with the lone pairs of electrons in antibiotics, thereby accelerating the oxidation process of antibiotics (56). This coordination reaction facilitates the transfer of electrons within the molecule, allowing antibiotic oxidation and degradation to occur even in natural environments. Divalent cations play a crucial role in microbial metabolism and catalytic reactions of enzymes (57). In this study, four divalent metal ions were selected to study their effects on *Bacillus* sp. DLY-11’s degradation of SMM. As shown in Fig. 2D, among these metal ions, overall, they enhanced the degradation effect, with Mg^2+^ > Mn^2+^ ≈ Ca^2+^ > Fe^2+^. From the degradation trends, experiments with MgSO_4_ and MnSO_4_ maintained a higher degradation efficiency in the first 16 h; especially, the MgSO_4_ group achieved a degradation rate of 84.1% after 24 h, significantly better than the FeSO_4_ group (46.2%) and the CaCl_2_ group (75.9%). This could be because Mg^2+^, as an important enzyme cofactor, plays a key role in microbial metabolism (58). Iron (Fe) is one of the indispensable elements in living organisms, participating in important cellular functions such as DNA biosynthesis (59). Adequate Fe^2+^ can promote DLY-11 degradation of SMM, but an excess of Fe^2+^ in the first 16 h may induce oxidative stress, slowing down the growth of the strain and affecting the initial degradation efficiency (60). However, the specific mechanisms of divalent cations in microbial degradation processes require further research.

Based on the above experimental results, adding MgSO_4_ was most effective for enhancing the DLY-11 strain’s degradation of SMM. Therefore, this study selected different concentrations of MgSO_4_ to observe its impact on SMM degradation (Fig. 2E). The results showed that with the increase in MgSO_4_ concentration, the degradation effect first decreased and then increased. When the MgSO_4_ concentration was 5 g/L, the best degradation efficiency within 48 h was reached at 94.4%. Mg^2+^ is an important metal element in living organisms, participating in energy metabolism, enzyme activity regulation, and various biochemical processes. Different concentrations of MgSO_4_ may affect key metabolic pathways in cells. It can be inferred that at lower MgSO_4_ concentrations, microorganisms might upregulate some gene expressions that promote biofilm formation and adhesion, negatively impacting the initial SMM degradation efficiency. However, as the MgSO_4_ concentration increases, these gene expressions may be downregulated while upregulating other gene expressions that favor SMM degradation (61).

### Optimization of degradation conditions for SMM degradative strain

#### Optimization of degradation conditions

Using the combination factor levels from each experimental group, SMM biodegradation experiments were conducted with the results shown in Table A.3. The ANOVA of the model is presented in Table 1. SMM degradation is the dependent variable. The mathematical relationship between the responses of three independent variables was evaluated using the following quadratic polynomial equation: Y1 = 97.43 + 1.50A − 0.2087B + 0.4438C + 0.215AB + 0.28AC − 0.4425BC − 0.7817A² + 0.2607B² − 0.3743C².

**TABLE 1:** Biodegradation products of SMM

Product ID	Molecular formula	*m/z*	Mass error (ppm)	Retention time (min)	Structure	Name
TP157	C_6_H_7_NO_2_S	157.0147	−0.2	12.553	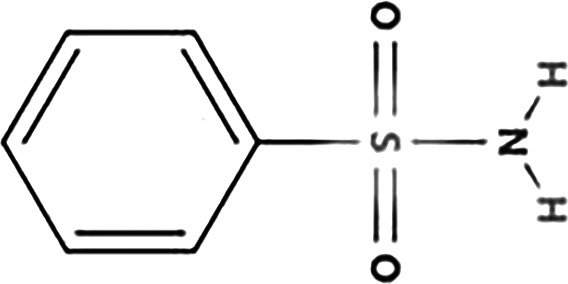	Benzenesulfonamide
TP172	C_6_H_8_N_2_O_2_S	172.0181	0.8	12.541	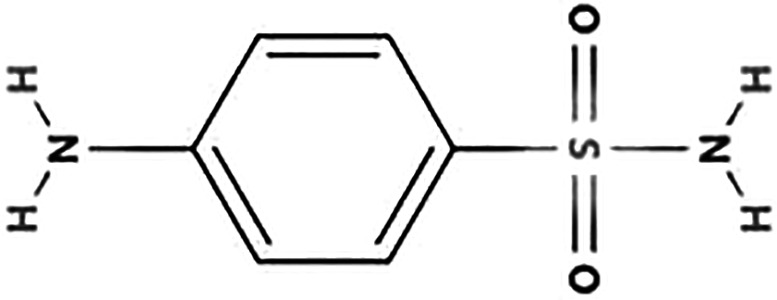	4-aminobenzenesulfonamide
TP187	C_10_H_9_N_3_O	187.2253	1.1	16.642	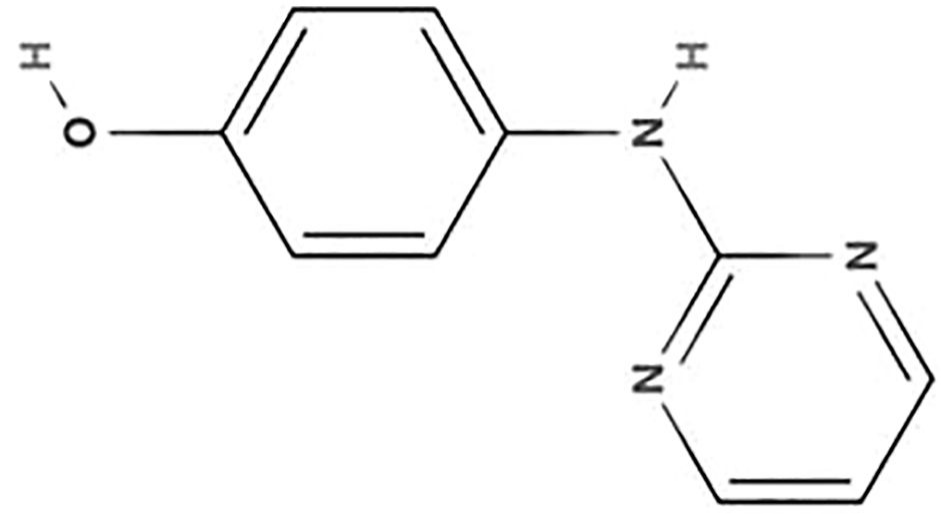	4-hydroxy-N-(pyrimidin-2-yl)benzene-1-amine
TP214	C_12_H_14_N_4_	214.2165	1.6	27.859	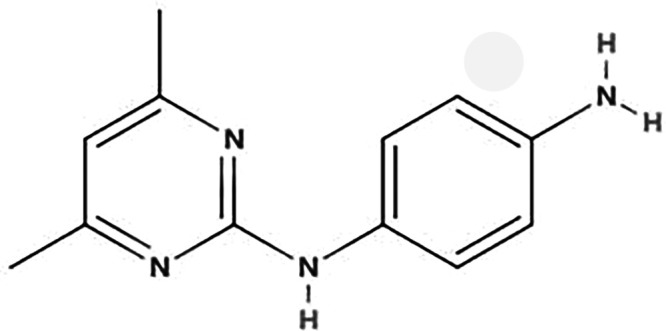	N-(4,6-dimethylpyrimidin-2-yl)benzene-1,4-diamine
TP245	C_12_H_12_O_2_N_4_	245.0966	−1.1	7.929	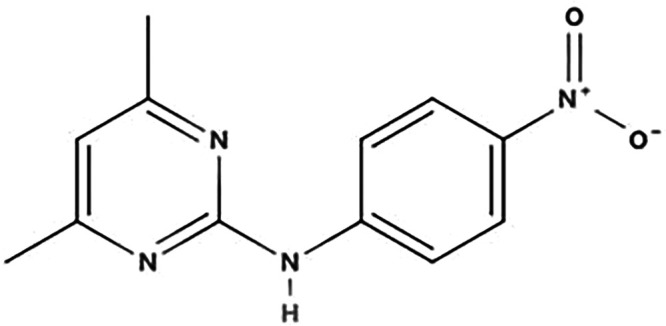	4-nitro-1-(4,6-dimethylpyrimidin-2-yl)benzene-amine
TP263	C_11_H_12_N_4_O_2_S	263.0971	2.2	7.616	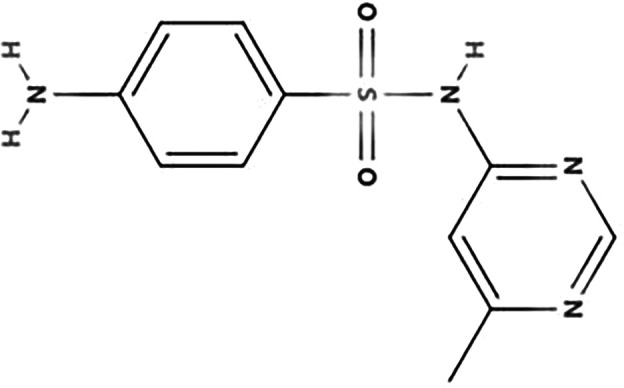	4-amino-N-(1-methyl-4,6-diazabenzene-3-yl)benzenesulfonamide

In the equation, Y1 represents the response of SMM degradation. The coded factor equation can be used to predict the response at given factor levels. By default, the high level of a factor is coded as +1, and the low level is coded as −1. By comparing the factor coefficients, the coded equation helps determine the relative impact of each factor.

The comparison between the predicted values and experimental values for the SMM degradation response is shown in Table 2. The results indicate that the overall model F-value is 44.57, with the corresponding *P*-value being much less than 0.0001, indicating a significant effect of the independent variables on the dependent variable, which means the model has stability and reliability, that is, the experimental model can well explain the changes in the response variable. The *R*^2^ (coefficient of determination) value reached 0.9828, and the adjusted *R*^2^adj was 0.9608, further enhancing the credibility of the model. Among all the factors, factor A (X1) has a very significant impact on the response variable, with its *P*-value less than 0.0001. Factor B (X2) also has a significant impact on the response variable, with its *P*-value being 0.0079. The *P*-value of factor C (X3) is 0.0671, approaching the significance level of 0.05, but has not yet reached statistical significance. The interaction analysis showed that no significant interactions of AB, AC, and BC were observed, with their *P*-values all greater than the significance level of 0.05. In addition, the *P*-value of C^2^ (X3^2^) is 0.9972, indicating that it has no significant impact on the response variable. Further analysis shows that the experimental model performs well in explaining the total variance. The *P*-value of the non-fit test is 0.98, indicating that the difference between the residuals of the model and the model variance is not significant. This means that the model may have some errors in predicting the response variable in specific areas but has a good overall fit. However, this study may have overlooked some minor factors. First, the *P*-value of factor C (X3) is close to the significance level, requiring further investigation of its impact on the response variable. Second, the interaction results did not reach statistical significance, suggesting that there may be some counteracting factors leading to this result. These minor factors require deeper research to better understand their impact. In summary, factors A (temperature) and B (pH) have significant impacts on the response variable, while factors C (MgSO_4_ concentration) and interactions (AB, AC, and BC) are statistically insignificant.

**TABLE 2:** Analysis of variance for SMM degradation^*a*^

Source	Sum of squares	df	Mean square	F-value	*P*-value	Significance
Model	69.57	9	7.73	44.57	<0.0001	Significant
A-X1	66.12	1	66.12	381.24	<0.0001	
B-X2	2.34	1	2.34	13.51	0.0079	
C-X3	0.8128	1	0.8128	4.69	0.0671	
AB	0.1849	1	0.1849	1.07	0.3362	
AC	0.0036	1	0.0036	0.0208	0.8895	
BC	0.0132	1	0.0132	0.0762	0.7904	
A²	0.0366	1	0.0366	0.2111	0.6598	
B²	0.055	1	0.055	0.3169	0.5911	
C²	0.000002368	1	0.000002368	0	0.9972	
Residual	1.21	7	0.1734			
Lack of fit	0.0494	3	0.0165	0.0566	0.98	Not significant
Pure error	1.16	4	0.2912			
Cor total	70.78	16				

^*a*^ *R*^2^ = 0.9828；*R*^2^adj= 0.9608.

### Response surface analysis of SMM

**Fig 3:**
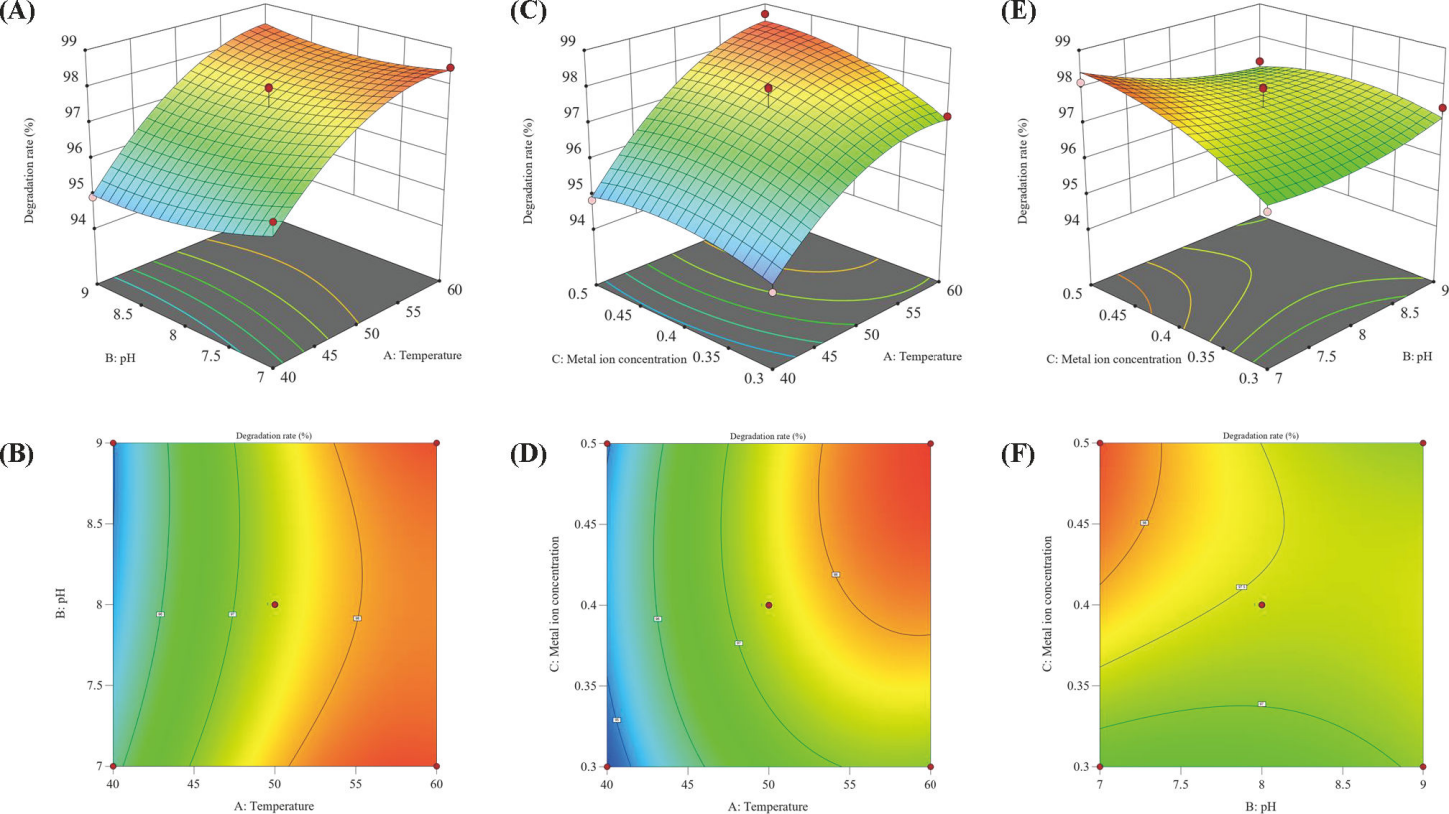
Response surface and contour plots of SMM degradation rates. Surface plot (**A**) and contour plot (**B**) of SMM degradation rate versus temperature (X1) and pH (X2); surface plot (**C**) and contour plot (**D**) of SMM degradation rate in relation to temperature (X1) and metal ion concentration (X3); surface plot (**E**) and contour plot (**F**) of SMM degradation rate versus pH (X2) and metal ion concentration (X3).

By observing the response of SMM degradation rate to different temperatures and pH values when the MgSO_4_ concentration is 0.4 g/L and the contour plots (Fig. 3A and B), it can be found that pH and temperature significantly affect the response values, but their interaction is not significant. Specifically, under the same pH conditions, increasing the temperature increases the removal rate of SMM. This indicates that temperature has a significant impact on the SMM degradation rate, and the response surface shows a slope that is lower on the left and higher on the right. Based on the results of the response surface analysis, the optimal temperature condition is approximately 60°C. The interaction of AC in Fig. 3C and D has an effect similar to that of AB, but under fixed temperature conditions, increasing the MgSO_4_ concentration results in a first decrease and then an increase in the SMM degradation rate. Increasing the concentration of MgSO_4_ under lower temperature conditions does not significantly improve the SMM degradation effect. However, as the temperature gradually increases, increasing the MgSO_4_ concentration significantly improves the SMM degradation effect. It is noteworthy that even at lower concentrations of magnesium ions, a temperature around 60°C can significantly improve the SMM removal rate, further demonstrating the importance of temperature in SMM degradation. The response surface and contour plots of pH and MgSO_4_ concentration at a temperature of 50°C (Fig. 3E and F) clearly show that an appropriate initial pH or high concentration of magnesium ions can promote SMM degradation. As the concentration of magnesium ions or the initial pH value increases, the SMM removal rate first increases and then decreases. These results indicate that adjusting the concentration of metal ions and pH can more effectively degrade SMM. The steepness plot in Figure B.2 shows that when the temperature reaches 59.1°C, pH is 7.10, and MgSO_4_ concentration is 0.45 g/L, the biological degradation rate of SMM reaches its peak. The predicted maximum SMM degradation rate is 98.8%. A validation experiment conducted under these conditions showed an actual degradation rate of 98.6%, which is very close to the predicted result, confirming the reliability of the model. Multiple studies have shown that *Bacillus thuringiensis* exhibits significant ability to degrade antibiotics. Zuo et al. (62) isolated the Bt strain HM-311 (CP040782–CP040785) from polluted soil in Xinjiang, which showed high resistance to various antibiotics such as lincomycin, tetracycline, rifampicin, furfural, fosfomycin, and amoxicillin. Zhou et al. (63) screened a *Bacillus thuringiensis* strain GIMCC1.817 that could effectively remove 77% of 1 µM erythromycin within 24 h, with 53% being completely degraded. Wen et al. (64) found that by converting the *Bacillus thuringiensis* strain H38 into immobilized bacteria, its removal rate of sulfadiazine (SM2) reached 98% within 36 h. In comparison, the *Bacillus thuringiensis* DLY-11 strain screened in this study could completely degrade 20 mg/L SMM within 48 h, with a degradation rate of 98.8%, demonstrating excellent antibiotic degradation performance. These results collectively confirm the great potential of *Bacillus thuringiensis* in degrading antibiotics. Given the high degradation efficiency of *Bacillus thuringiensis* DLY-11 toward SMM and its excellent environmental adaptability, we proposed several potential application scenarios: (i) Preparation of single or composite degradative inoculants for aerobic composting treatment of livestock and poultry manure. (ii) Treatment of wastewater from farms and slaughterhouses containing SMM residues. (iii) Treatment of industrial wastewater from SMM production enterprises and medical wastewater. (iv) Remediation of SMM-contaminated farmland and soil around farms and slaughterhouses. In summary, by optimizing the physicochemical properties of SMM and the physiological demand conditions of *Bacillus* sp. DLY-11, we achieved efficient degradation, providing an effective solution for managing SMM pollution.

### Possible degradation mechanisms of strain DLY-11 on SMM

**Fig 4:**
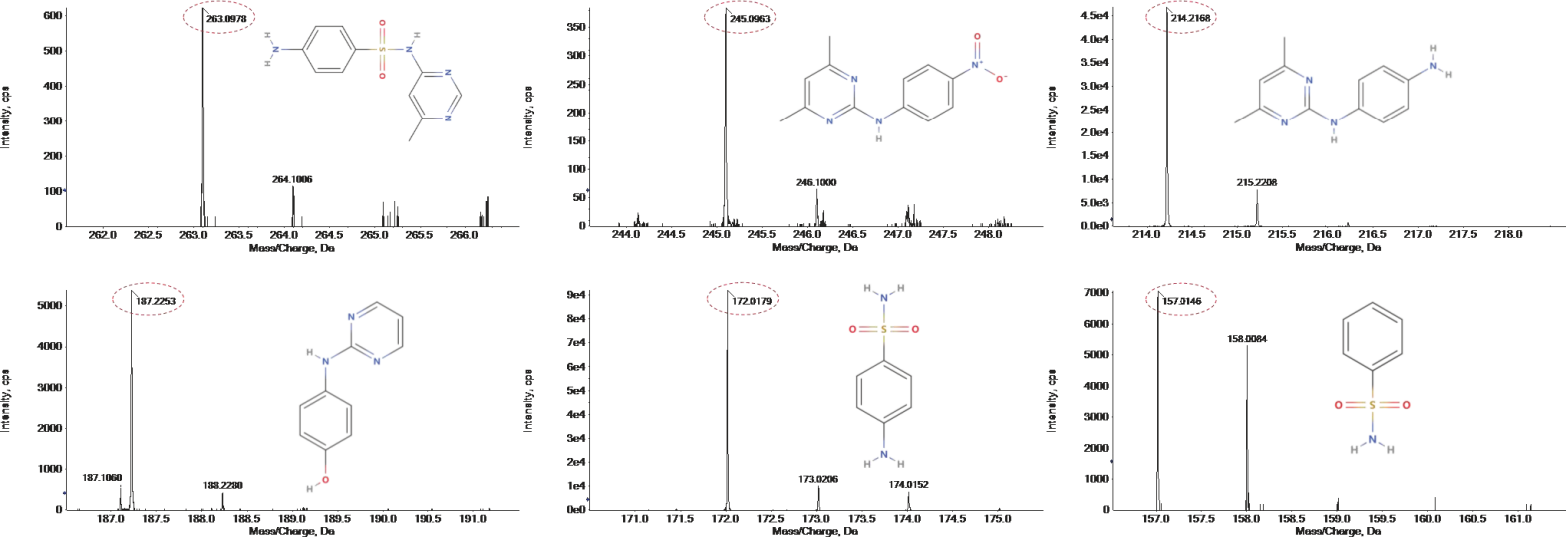
Mass spectrometry of SMM biotransformation products.

**Fig 5:**
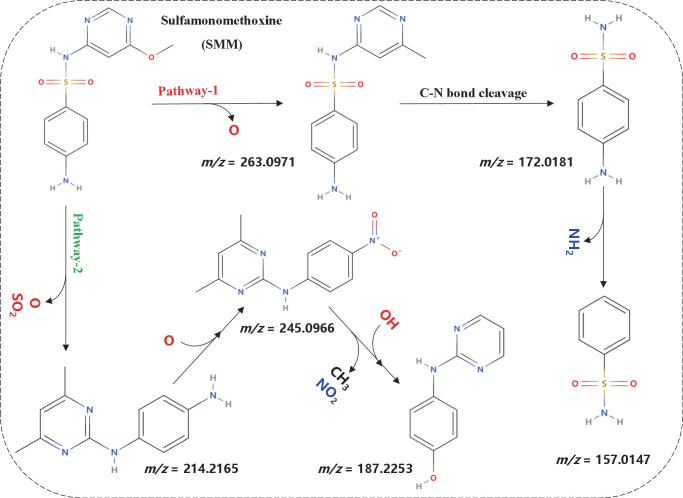
Biodegradation pathway of SMM by *Bacillus thuringiensis* DLY-11.

According to Fig. 4 and Table 1, significant peaks of compounds with different molecular weights are shown, indicating the products generated by strain DLY-11 after degrading SMM. By analyzing the retention time and mass of fragment ions, we identified six potential transformation products (TPs) of SMM, each with different *m/z* values: 263.0971, 245.0966, 214.2165, 187.2253, 172.0181, and 157.0147. The chemical formula of TP263 is C_11_H_12_N_4_O_2_S (O from SMM), suggesting it may be 4-amino-N-(1-methyl-4,6-diazabenzene-3-yl)benzenesulfonamide, indicating it could be a product of the reduction reaction of SMM. TP172, which is 4-aminobenzenesulfonamide, was detected and confirmed as a transformation product of SMX degradation by *Pseudomonas psychrophila* HA-4 strain (65). Similarly, SMX-TP172 (4-aminobenzenesulfonamide) was also detected in the degradation of SMX by aerobic *Vibrio ohioensis* MR-1 and *Shewanella* sp. MR-4 strains (66). In our study, we also detected the presence of 4-aminobenzenesulfonamide (TP172). The chemical formula of TP157 is C_6_H_7_NO_2_S (–NH_2_ from TP172), suggesting it may be a product obtained after TP172 undergoes a deamination reaction. The chemical formula of TP214 is C_12_H_14_N_4_ (–SO_2_ from SMM), indicating it may be a product obtained after SMM releases SO_2_ (67). The chemical formula of TP245 is C_12_H_12_O_2_N_4_, and it is speculated to be 4-nitro-1-(4,6-dimethylpyrimidin-2-yl)benzene-amine, indicating that nitrification was observed in our study, and TP245 may be a product formed after TP214 undergoes a nitrification reaction. TP245 is also a degradation product that has not appeared in previous studies (68, 69). The chemical formula of TP187 is C_10_H_9_N_3_O, and it is speculated to be 4-hydroxy-N-(pyrimidin-2-yl)benzene-amine, indicating it may be a product obtained after TP245 undergoes denitrification, demethylation, and hydroxylation reactions. Based on the degradability of SMM and the detected intermediate products, this study proposes two possible degradation pathways (as shown in Fig. 5).

#### Pathway 1

SMM first undergoes a reduction reaction to generate 4-amino-N-(1-methyl-4,6-diazabenzene-3-yl)benzenesulfonamide (*m/z* 263.0971). This molecule is then converted to 4-aminobenzenesulfonamide (*m/z* 172.0181) via C-N bond cleavage. Subsequently, 4-aminobenzenesulfonamide (*m/z* 172.0181) further undergoes deamination to generate benzenesulfonamide (*m/z* 157.0147).

#### Pathway 2

SMM undergoes methylation, reduction, and release of SO_2_ to produce N-(4,6-dimethylpyrimidin-2-yl)benzene-1,4-diamine (*m/z* 214.2165). This process has been reported in the studies by Li et al. (68) on the biological degradation of sulfamonomethoxine in a granular sludge system and by Yang et al. (70) on the use of nitrifying bacteria to promote irreversible conversion of sulfamonomethoxine. Previous studies have shown that oxidative reactions based on SO_4_^•-^ can convert sulfonamide compounds containing a six-membered heterocycle (such as Sulfamonomethoxine) into corresponding SO_2_ expulsion products (71, 72). Subsequently, the amino groups on the benzene ring of N-(4,6-dimethylpyrimidin-2-yl)benzene-1,4-diamine (*m/z* 214.2165) may be oxidized to nitro groups, generating 4-nitro-1-(4,6-dimethylpyrimidin-2-yl)benzene-amine (*m/z* 245.0966). Hydroxylation has been reported as an effective pathway for degrading quinoline groups, typically occurring in regions of higher electron cloud density. Since the C = N bond can reduce the electron cloud density of the ring and increase the electron density of the benzene ring, hydroxylation is more likely to occur on the benzene ring (25). Finally, 4-nitro-1-(4,6-dimethylpyrimidin-2-yl)benzene-amine (*m/z* 245.0966) undergoes hydroxylation, demethylation, and denitrification to produce 4-hydroxy-N-(pyrimidin-2-yl)benzene-amine (*m/z* 187.2253). This pathway is a novel SMM degradation pathway not previously reported in the literature.

These degradation pathways reveal the types of reactions that may occur during the degradation of SMM by strain DLY-11, including C-N bond cleavage, hydroxylation, SO_2_ release, reduction, methylation, deamination, demethylation, oxidation, and denitrification. Among these, C-N bond cleavage and SO_2_ release may be key initial steps. Based on the observed reaction processes (such as C-N bond cleavage, deamination, hydroxylation, etc.), several enzymes are likely to be crucial for the SMM degradation process: (i) monooxygenases or dioxygenases (20), involved in the initial hydroxylation of SMM; (ii) deaminases (73), responsible for the removal of amino acids; (iii) sulfotransferases (74), responsible for SO_2_ release and sulfur atom transfer. These enzymes may be located intracellularly, in the periplasm, or extracellularly, working together in the degradation of SMM.

Although a comprehensive genomic study has not been conducted, based on existing literature and experimental observations, the following genes are likely to be related to SMM degradation: (i) genes related to nitrogen metabolism (75), such as those involved in the deamination process; (ii) genes related to sulfur metabolism (75), such as those involved in SO_2_ release and sulfur atom transfer; and (iii) genes encoding these key enzymes, such as monooxygenases and halogenases. SMM degradation primarily focuses on the sulfonamide group, converting it to nontoxic or low-toxic products through microbial metabolism. In addition, the hydrolysis products of amide bonds and sulfonic acid groups are also evident in the mass spectrometry results and further degrade into simpler chemical substances. Oxidative reactions can increase the molecular weight of certain groups, enhancing water solubility but reducing biological activity. Reduction reactions assist in the further decomposition of sulfonamides by adjusting the oxidation states of functional groups. Meanwhile, the generation of low-molecular-weight benzenesulfonamide (*m/z* 157.0147) and 4-hydroxy-N-(pyrimidin-2-yl)benzene-1-amine (*m/z* 187.2253) significantly reduces the toxicity and environmental risk of these products.

### Conclusion

In this study a new strain, *Bacillus* sp. DLY-11, with significant SMM degradation capabilities, was isolated from swine manure compost. An RSM based on Box-Behnken design was used to optimize its degradation conditions. The results showed that under conditions of a 5% inoculation volume, a temperature of 59.1°C, a pH value of 7.10, and a MgSO_4_ concentration of 0.45 g/L, strain DLY-11 could degrade 20 mg/L of SMM to 98.8% within 48 h. These optimized conditions significantly improved the SMM degradation efficiency. Product analysis identified six potential transformation products and proposed two possible biological degradation pathways for SMM, including reactions such as C-N bond cleavage, hydroxylation, SO_2_ release, reduction, methylation, deamination, demethylation, oxidation, and denitrification. Notably, we discovered a novel degradation pathway not previously reported in the literature. This study not only provided a new strain resource for efficiently degrading SMM but also optimized the relevant degradation conditions and revealed new degradation mechanisms. These findings fill the knowledge gap regarding bacterial degradation pathways of SMM and provide theoretical and technical support for the biological remediation of antibiotic pollutants in livestock and poultry farming. This research helps alleviate the problem of antibiotic pollution, protecting the environment and human health. Future studies can further explore the molecular mechanisms of strain DLY-11 in degrading SMM, particularly the identification of key enzymes and functional genes, to gain a deeper understanding of the roles of microorganisms in the SMM degradation process.

### Highlights

- A novel *Bacillus *sp., DLY-11, was derived from swine manure composting.
- *Bacillus *sp. DLY-11 efficiently degraded SMM, achieving 98.8% degradation in 48 h.
- A novel degradation pathway for SMM was proposed.
- C-N bond cleavage, hydroxylation, and SO_2_ release were the main degradation mechanisms.

## Data Availability

All data generated or analyzed during this study are included in this published article.

## SUPPLEMENTAL MATERIAL

Graphical abstract aem.01329-25-s0001.tiff Visual diagram of the study.

Supplemental material aem.01329-25-s0002.doc Tables S1 to S4, Fig. S1 and S2, and supplemental methods.

ASM does not own the copyrights to Supplemental Material that may be linked to, or accessed through, an article. The authors have granted ASM a non-exclusive, world-wide license to publish the Supplemental Material files. Please contact the corresponding author directly for reuse.
