# Supplementary figures and images for: Optimization of degradation conditions and elucidation of novel biodegradation pathways for sulfamonomethoxine by a novel Bacillus strain
Source: Appl Environ Microbiol. 2025 Aug 12;91(9):e01329-25. doi: 10.1128/aem.01329-25 (PMC12442388; doi:10.1128/aem.01329-25)

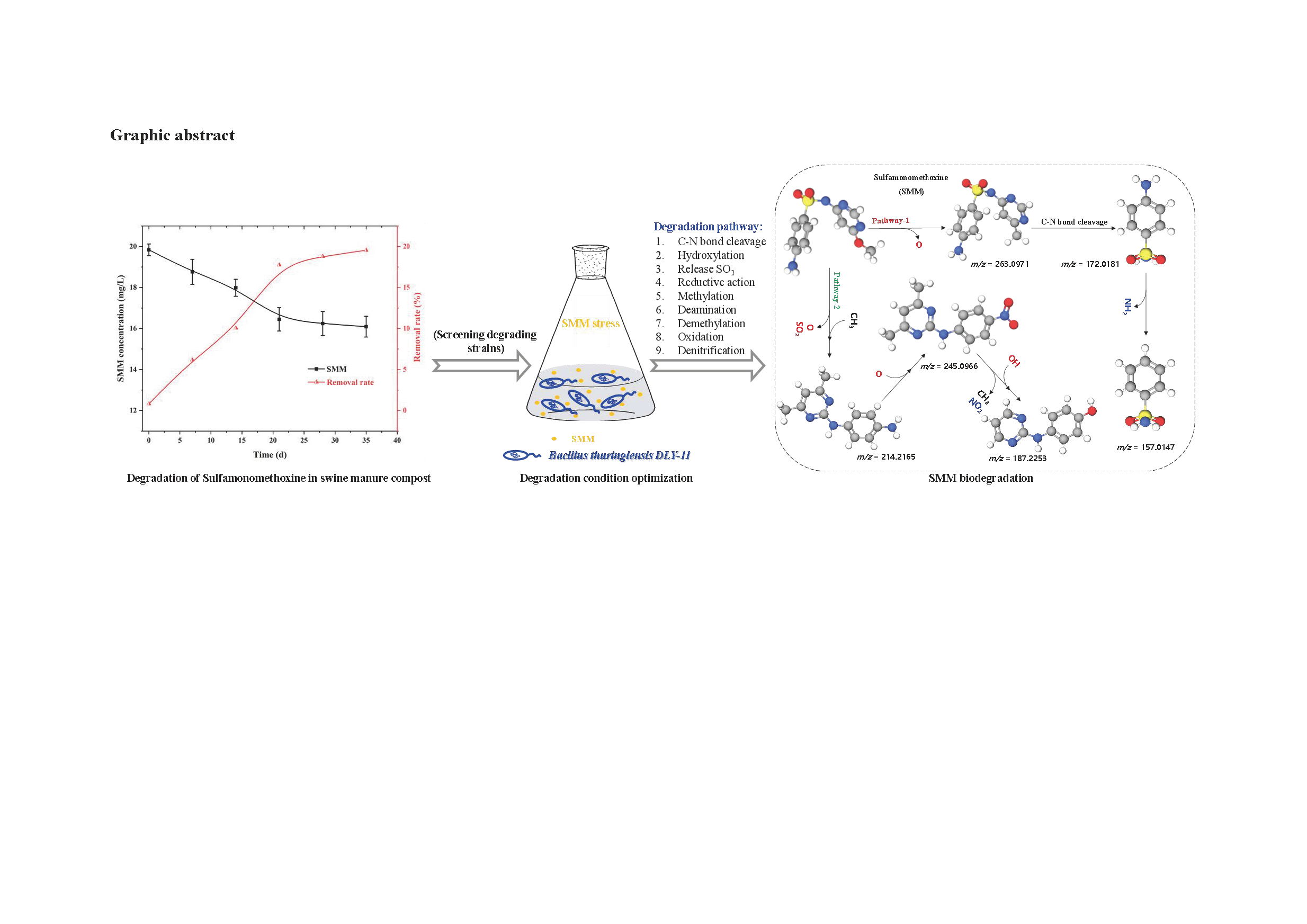

Supplement: Graphical abstract — Visual diagram of the study. [file aem.01329-25-s0001.tiff]
