# Supplementary material for: Optimization of degradation conditions and elucidation of novel biodegradation pathways for sulfamonomethoxine by a novel Bacillus strain
Source: Appl Environ Microbiol. 2025 Aug 12;91(9):e01329-25. doi: 10.1128/aem.01329-25 (PMC12442388; doi:10.1128/aem.01329-25)
Supplement: Supplemental material — Tables S1 to S4, Fig. S1 and S2, and supplemental methods. [file aem.01329-25-s0002.doc]

**Supplementary Material**

**Optimization of Degradation Conditions and Elucidation of Novel Biodegradation Pathways for Sulfamonomethoxine by a Novel *Bacillus* Strain**

**Xiujuan Wang^a, b^, Jingtong Li^f^, Chunyan Chen^e^, Zifeng Luo^a,b, *^, Yuwan Pang^d^, Hongxing Tu^a, b^, Xiaojun Lin^a, b, c^, Cuifen Long^g^, Qianyi Cai^a, b^, Zebin Wei^a^, Jinrong Qiu^a, b^**

1. *South China Institute of Environmental Sciences, MEE, Guangzhou, 510655, Guangdong, China*
2. *State Environmental Protection Key Laboratory of Water Environmental Simulation and Pollution Control, Guangzhou, 510655, Guangdong, China*
3. *College of Natural Resources and Environment, South China Agricultural University, Guangzhou 510642, China*
4. *Institute of Agricultural Resources and Environment, Guangdong Academy of Agricultural Sciences, Guangzhou 510640, China*
5. *Jinan University, Guangzhou 511443, China*
6. *College of Agriculture, Yangtze University, Jingzhou, 434025, Hubei, China*
7. *Guangzhou Municipal River and Channel Monitoring Center, Guangdong Province, Guangzhou 510650, China*

**Appendix A**

**Table A.1** Primer Information

| Detection fragment | Primer name | Primer sequence | Fragment size |
| --- | --- | --- | --- |
| Bacterial 16S | 27F | AGAGTTTGATCCTGGCTCAG | 1500bp |
|  | 1492R | TACGGCTACCTTGTTACGACTT |  |
|  | V4-515F | GTGCCAGCAGCCGCGGTAA |  |
|  | V4-806R | GGACTACCAGGGTATCTAA |  |
| Fungal 18S | ITS1 | TCCGTAGGTGAACCTGCGG | 400-800bp |
|  | ITS4 | TCCTCCGCTTATTGATATGC |  |

**Table A.2** Factors and Levels Box-Benhnken Design Table

| Factor | Factor levels and codes | | |
| --- | --- | --- | --- |
|  | -1 | 0 | 1 |
| X1 | 40 | 50 | 60 |
| X2 | 7 | 8 | 9 |
| X3 | 0.3 | 0.4 | 0.5 |

**Table A.3** The RSMs for the three experimental variables of SMM are coded units and corresponding natural values

| No. | Natural variables | | | Coding variables | | |
| --- | --- | --- | --- | --- | --- | --- |
|  | X1 | X2 | X3 | X1 | X2 | X3 |
| 1 | 60 | 8 | 0.3 | 1 | 0 | -1 |
| 2 | 60 | 8 | 0.5 | 1 | 0 | 1 |
| 3 | 50 | 9 | 0.3 | 0 | 1 | -1 |
| 4 | 50 | 8 | 0.4 | 0 | 0 | 0 |
| 5 | 40 | 7 | 0.4 | -1 | -1 | 0 |
| 6 | 50 | 8 | 0.4 | 0 | 0 | 0 |
| 7 | 50 | 7 | 0.5 | 0 | -1 | 1 |
| 8 | 50 | 8 | 0.4 | 0 | 0 | 0 |
| 9  10 | 60  50 | 9  9 | 0.4  0.5 | 1  0 | 1  1 | 0  1 |
| 11 | 40 | 9 | 0.4 | -1 | 1 | 0 |
| 12 | 60 | 7 | 0.4 | 1 | -1 | 0 |
| 13 | 40 | 8 | 0.5 | -1 | 0 | 1 |
| 14 | 50 | 8 | 0.4 | 0 | 0 | 0 |
| 15 | 50 | 7 | 0.3 | 0 | -1 | -1 |
| 16 | 40 | 8 | 0.3 | -1 | 0 | -1 |
| 17 | 50 | 8 | 0.4 | 0 | 0 | 0 |

Note: Validation experiment: To verify the accuracy and effectiveness of the model, two parallel experiments were conducted under the optimized SMM degradation conditions. The corresponding response values are taken as the average of the two experimental results to verify whether the experimental values are consistent with the predicted values.

**Table A.4** Conditions for MRM determination of sulfonamide antibiotics

| Compound | Parent ion | Daughter ion | CE(eV) | DP(V) | Retention time (min) |
| --- | --- | --- | --- | --- | --- |
| SMM | 311.1 | 156.1*,108.1,92.1 | 23,30,32 | 70 | 4.1 |

**Appendix B**

**Fig. B.1** Aerobic composting device schematic diagram


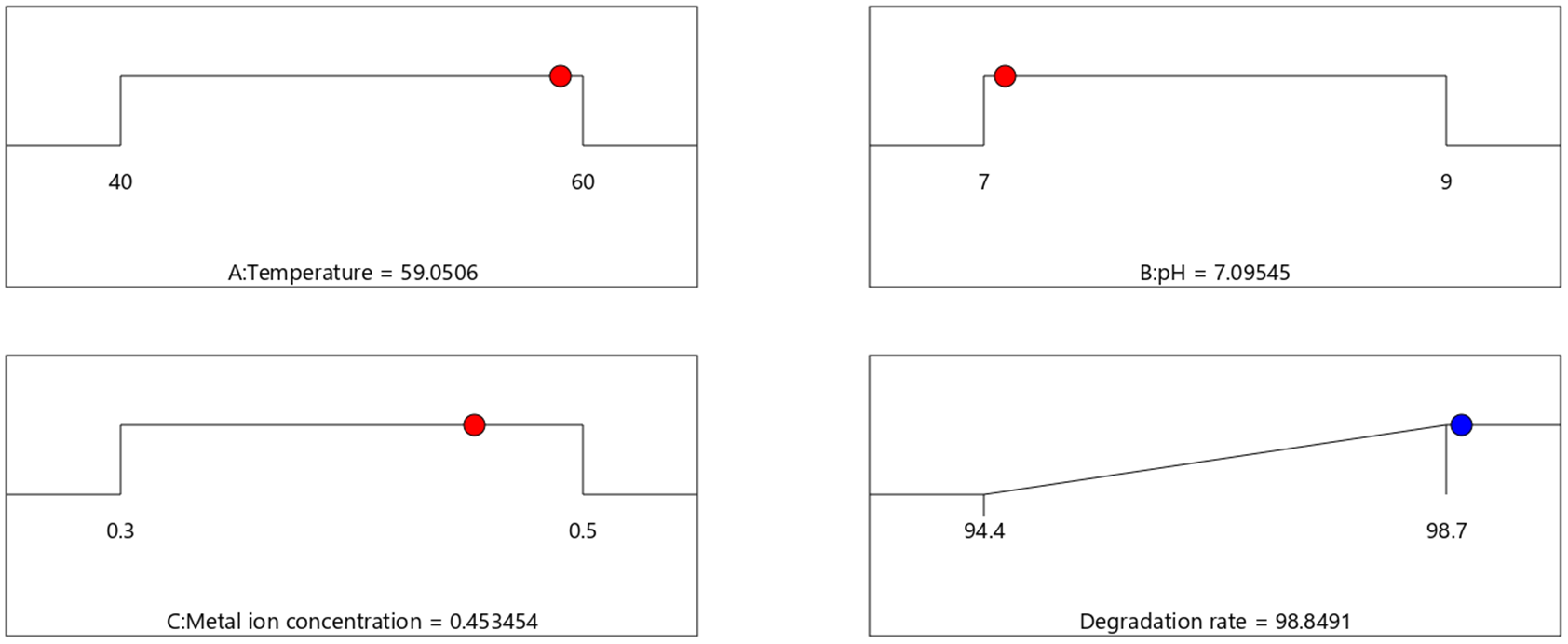


**Fig. B.2** Degradation SMM optimal condition feasibility slope diagram

**Appendix C**

Preparation of Standard Solutions

Accurately weigh 0.0050 g of each of four antibiotic standards, dissolve in pure methanol, and make up to 10.0 ml in volumetric flasks, preparing standard stock solutions at a concentration of 500 mg/L. Store these solutions at -18℃ in the dark (not exceeding one month). The standard curve is prepared by diluting these stock solutions. Here's the specific procedure: Transfer 2.0 ml of each antibiotic stock solution into separate 20.0 ml volumetric flasks to prepare a mixed standard solution of 50 mg/L. Then, transfer 0.5, 1.0, 2.0, 3.0, and 4.0 ml of the mixed standard solution into five 10.0 ml volumetric flasks, and diluted with pure methanol, to obtain standard series concentrations of 2.5, 5.0, 10.0, 15.0, and 20.0 mg/L. Store these at 4℃ in the dark and should be utilized on the same day.

**Appendix D**

Parameters of Composting Apparatus

In the experiment, a PVC barrel with a diameter of 100 cm and height of 80 cm was used as the composting container. A ventilation pipe is connected at the bottom of the barrel, with a layer of gravel placed on top to ensure even ventilation. A mesh cloth is laid over the gravel, and compost material is placed on the mesh. The ventilation pipe is connected to a blower to control ventilation rate via a valve. The blower is connected to a timer to control ventilation time. At the top of the compost material, a woven bamboo sieve is placed, covered with a layer of rice straw for insulation and moisture retention.

**Appendix E**

Screening and Isolation of SMM Efficient Degradation Strains

Matured material from aerobic composting of pig manure was used as the inoculum enrichment medium. The specific process is as follows: First, enrichment culture is conducted. Weigh 10 g of sterilized and quartered experimental sample into a sterilized triangular flask, add an appropriate amount of sterilized distilled water, shake to suspend for about 5 minutes, then naturally settle for 1 hour. Take 1 ml of the supernatant and spread it on enrichment medium containing SMM, and culture at 30℃. Next, preliminary screening and isolation of strains are conducted. Multiple isolations are performed on colonies with similar color and size on the enrichment medium until pure under a microscope. Then, strain acclimatization is conducted. The selected bacteria are inoculated into selection medium containing SMM in a shaking incubator, starting from a concentration of 100 μg/mL with increments of 100 μg/mL as gradients, at 30℃ and 80 rpm, with 7 days per acclimatization cycle and a total of 4 cycles. Finally, re-screening of strains is conducted. A certain amount of acclimatized liquid is inoculated into selection medium, shaken at 30℃ and 80 rpm. A blank control without inoculation is used to eliminate factors such as hydrolysis that may reduce SMM. Samples are taken at regular intervals to determine SMM content, calculate degradation rate, and thus screen out highly active dominant strains.

**Appendix F**

SEM Testing Procedure

Critical point drying sample preparation involves taking samples from glutaraldehyde, followed by gradient dehydration in 50%, 70%, and 100% ethanol for 10 minutes each. After dehydration, samples are taken out with 100% ethanol and placed into a supercritical drying machine for critical point drying. Drying time is approximately 1 hour. After drying, samples are taken from the sample chamber of the critical point dryer, fixed on a sample stage with conductive tape, gold-sputtered, and then tested.

**Appendix G**

Analysis of 16S rRNA Sequences and Strain Genetics

Commercial kits are used to extract bacterial genomic RNA, with RNA as a template to amplify specific segments. Universal primers for amplifying 16S rRNA genes are synthesized by Guangdong MegBioTech Co., Ltd. PCR reaction system (50 μL): 10× PCR buffer 5 μL, dNTPs (25 mmol/L) 1.0 μL, upstream and downstream primers (20 mmol/L) each 1.0 μL, template RNA 1.0 μL, Mg^2+^ (25 mmol/L) 3.0 μL, TaqRNA polymerase 0.5 μL, and supplemented with ultrapure water to 50 μL. The reaction parameters were as follows: pre-denaturation at 94°C for 5 min, denaturation at 94°C for 40 s, annealing at 60°C for 40 s, extension at 72°C for 1 min, for 30 cycles, followed by a final extension at 72°C for 10 min, and then stored at 4°C for 10 min. PCR amplification products were analyzed by 0.8% agarose gel electrophoresis for validation, followed by sequencing of the samples. Primer information is detailed in Table A.1 The obtained 16S rRNA sequences were compared for homology analysis using the NCBI GenBank database (www.ncbi.nlm.nih.gov). Homology and genetic distances were analyzed using Bioedit, and relevant bacterial 16S rRNA sequences were downloaded. All sequences were aligned and trimmed using ClustalX1.8.3. A phylogenetic tree was constructed using the neighbor-joining method in MEGA7.0 for systematic evolutionary analysis.

**Appendix H**

Box-Benhnken Design

BBD is a second-order design based on three-level incomplete factors. Compared to other response surface design methods, it generates a total of 9 coefficients with fewer runs [1]. To estimate adjusted parameters for a quadratic response surface model with N variables, the study included a BBD of 17 runs with three factors at three levels and three replicates at center points. These serve as measures of data consistency and repeatability and have been proven useful. Use F-test significance (0.05) and lack of fit test significance (non-significant) to determine adequacy of the fitted model [2]. Therefore, this model can also be used to optimize the conditions for SMM biodegradation within the range of test parameters. Each independent variable (i.e., low, medium, and high) was assigned values of -1, 0, and +1, as shown in Table A.2. The coded units of RSM for the three experimental variables and their corresponding natural values are listed in Table A.3.

**Appendix I**

An analytical method using high-performance liquid chromatography (HPLC, Alliance e2695, Americas) was employed to determine SMM content in samples, and LunaC18column (5 μm, 2.0 mm×150 mm) was used with a mobile phase of 0.01 mol/L oxalic acid solution and methanol at a flow rate of 1.0 mL/min, with a sample injection volume of 5 μL. The column temperature was maintained at 30°C, and detection was performed at a wavelength of 268 nm. For analysis of SMM metabolites, a method employing liquid chromatography-mass spectrometry (LC-20AD, Shimadzu) coupled with a high-resolution hybrid quadrupole time-of-flight mass spectrometer (AB SCIEX X500R Q-TOF) was used. Using LC-20AD separation with a Waters LC BEH C18 column (2.1×50mm×1.7μm), a sample injection volume of 10 μM, and a mobile phase consisting of 0.1% formic acid (A) and methanol (B), and the elution gradient was as follows: initial, 5% B; 0–1 min, 25% B; 1–7 min, 35% B; 7–13 min, 95% B; 13–15 min, 95% B; 15–15.1 min, 5% B held for 3 min, at a flow rate of 0.3 mL/min. Mass spectrometry was performed on an AB SCIREX QTRAP 5500+MS/MS system. Preliminary analysis was conducted in EMS-IDA-EPI mode (enhanced mass spectrometry scanning-enhanced product ion scanning) under the following conditions: ion source, electrospray ionization [3]; scan mode, positive ion scan; detection mode, multiple reaction monitoring (MRM); ESI voltage, 5500 V; nebulizer gas pressure (GS1), 50.0 psi; auxiliary gas flow rate (GS2), 50.0 psi; curtain gas pressure (CUR), 40.0 psi; collision gas (CAD), 7.0 psi; ion source temperature [4], 500°C; scan time, 10 ms; collision cell exit voltage (CXP), 11.0 V; collision cell entrance voltage (EP), 10.0 V; qualitative ion pairs, quantitative ion pairs, collision energy (CE), and declustering potential [5]（Table A.4）。

**References**
